# Generative modelling of continuous feature foraging reveals probabilistic representations of target distributions

**DOI:** 10.3758/s13414-026-03227-6

**Published:** 2026-03-18

**Authors:** Jennifer C. Magerl Fuller, Árni Kristjánsson, Alasdair Clarke, Árni Gunnar Ásgeirsson

**Affiliations:** 1 Faculty of Psychology, Nýi Garður, University of Iceland, Reykjavík, Iceland; 2 Faculty of Psychology, University of Essex, 2.719 Colchester Campus, Colchester, UK; 3 Faculty of Psychology, University of Akureyri, Akureyri, Iceland

**Keywords:** Feature distribution learning, Bayesian modelling, Visual perception, Visual attention

## Abstract

To successfully orient ourselves within noisy visual environments, we must focus our attention on items of importance, ignoring sources of distraction. This selective attending is typically thought to be facilitated by templates, tuned towards current goals. However, in real-world scenes, the appearance of objects, such as their colour or luminance, varies greatly due to perceptual interpretation and environmental factors. Therefore, tuning attentional templates probabilistically may be more efficient than tuning them to precise values. This seems particularly important during continuous tasks, that require the selection of multiple objects which share certain properties. We investigated the effects of variability in target identity, using a novel foraging task. Participants (N = 15) had to continuously select 30 target objects, drawn from a truncated Gaussian colour distribution, sampled from a linearized space of 48 isoluminant hues. We adapted a generative model and applied it to the data, within a Bayesian multilevel framework. The model characterizes foraging as a sampling process without replacement and allows us to break foraging down into behavioural patterns that influence individual's target selection, independent of the number of targets present. The modelling results demonstrate increased likelihood of selection of more probable colour values in the scene. This likelihood maps onto the underlying probability distribution, illustrating how observers can acquire knowledge of the distribution's properties through foraging, beyond just the summary statistics.

## Introduction

Selective visual attention involves narrowing our focus down onto a subset of elements that are available within the visual environment (Desimone & Duncan, 1995; Neisser, 1967). A common theoretical view is that *attentional templates* are formed during visual search, which are tuned towards our current goals (Bundesen, 1990; T. Kristjánsson et al., 2018; Woodman et al., 2013), biasing the system towards objects with template-matching attributes.

For example, when you search for a friend in a crowd who is wearing an orange hat, your visual attention would be tuned to orange. However, our goals are not always so narrow, and in a given visual scene there can be many relevant targets. The complexity of naturalistic visual scenes makes it impractical to tune templates to exact values (such as exact luminance or colour), due to the variability in environmental circumstances and perceptual interpretation. Tuning templates to a range of values would seem to be more effective, such as in probabilistic tuning, where the variability in environmental statistics is assimilated (Kristjánsson, 2022). Recent evidence shows how the visual system learns to represent statistical variation through repeated exposure to environmental statistics (Chetverikov et al., 2017a), in what can be termed "probabilistic attentional selection" (Kristjánsson, 2022; Tanrikulu et al., 2021). Our aim was to determine whether attentional selection reflects the learning of the underlying probability density of target objects and to address the unresolved issue of the specificity of attentional templates (Bravo & Farid, 2009; Malcolm & Henderson, 2009). Additionally, we investigated whether these templates are tuned to precise feature values (colour in the current project) or are more broadly tuned.

A naturalistic example of template tuning would be a task such as picking blueberries. Factors such as viewing position, orientation relative to a light source, and whether the berries are in the shade or direct sunlight result in substantial variation in actual luminance and colour values. However, our perceptual interpretation of the hue of the berries remains relatively constant. This constancy raises questions about the mechanisms guiding attention and the tuning of efficient attentional templates. Tuning attention to a range of values might prove more effective in guiding selection, rather than tuning to singular feature values. The visual system might become "primed" to values in the environment, such as the colour range of the blueberries, thereby developing a representation of the underlying colour distribution. The central premise is that such representations are probabilistic and continuously updating, according to Bayesian learning (Kristjánsson,  2022). This probabilistic conception of perceptual representation is supported by evidence from neurophysiology, such as neural population encoding (Lehky & Sejnowski, 1999; Walker et al., 2020), and theoretical neuroscience, which shows that such encoding is necessarily probabilistic (Averbeck et al., 2006; Ma & Jazayeri, 2014). Additionally, psychophysical evidence demonstrates that attentional priming operates probabilistically (Chetverikov et al., 2017a, 2017b, 2017c; Hansmann-Roth et al., 2021).

## Attentional priming

Attentional priming affects the processing of feature identity (Maljkovic & Nakayama, 1994), and the encoding of feature probability distributions, which bias attentional selection (Chetverikov et el., 2017b). Attentional priming is widely accepted to be a mechanism which guides attention towards relevant current goals (Á. Kristjánsson & Ásgeirsson, 2019; Suresh & Wolfe, 2021) while extraneous, irrelevant information is ignored (Theeuwes, 2010). Such priming can apply to assembled objects (Ásgeirsson & Kristjánsson, 2011) or features (Maljkovic & Nakayama, 1994), where the accuracy of the template gradually increases over time and repeated exposure. Since targets in natural environments are unlikely to have precisely identical hues, attentional templates tuned to probabilities would seem to be more efficient (Chetverikov et al., 2020; Kristjánsson, 2022). Following this logic, it would seem likely that attention would be guided towards target features represented at higher probabilities within the visual scene, and that this would be reflected in the pattern of selections.

Recent studies have addressed such probabilistic templates. Chetverikov et al. (2016) used role-reversal effects of targets to distractors (Á. Kristjánsson & Driver, 2008) to show that feature probability predicts the size of the role-reversal effects. Chetverikov et al. (2016) found that response time curves reflected distribution shape, not just summary statistics, as has been previously suggested (Alvarez, 2011; Cohen et al., 2016). Probabilistic target templates have been less well-studied, but Hansmann-Roth et al. (2022) tested the learning of target distributions in a paradigm with a single target amongst two distractors. Observers performed a discrimination task on a target drawn from linearized colour space, from a distribution with a specific shape, and distractors drawn from another section of colour space. The larger the number of consecutive searches, the better response times mapped onto the target colour distribution shape showing how observers learned the distribution characteristics over a sequence of adjacent trials, even when only one target exemplar was presented on each trial. Furthermore, search times were significantly slower for targets drawn from the distribution tails, and this was more pronounced for Gaussian than uniform target distributions, indicating that the distribution properties were learned.

## Analysing multitarget foraging tasks with continuous feature values

Multitarget foraging experiments typically sort targets into discrete feature value categories (Cain et al., 2012; Fougnie et al., 2015; Hills et al., 2012, 2013; Á. Kristjánsson et al., 2014, 2019; Thornton et al., 2022; Wolfe, 2013; Wolfe et al., 2016, 2019) such as the colours orange and green. Sometimes targets are sorted into multiple discrete feature categories, as in conjunction foraging (Á. Kristjánsson et al., 2014, 2020a, 2020b; Wolfe et al., 2019). In these tasks, a target can be identified through a single categorical feature, e.g. the colour orange, or a conjunction of features (e.g., the colour orange and a triangular shape). Foraging tasks where continuous target feature values are used, may provide a more ecologically valid depiction of foraging behaviour.

Despite the obvious utility of foraging paradigms for investigating visual attention, there are some limitations to how such tasks have typically been analysed. A key concern are the factors which influence sequential target selection; the process by which a particular target is selected from a set of alternate candidates (Tagu & Kristjánsson., 2022b; Wolfe et al., 2017, 2019). Clarke et al. (2022a, 2022b) modelled the ways in which foraging behaviour can be broken down into particular performance patterns, such as a preference to stick to the same target type, and a preference for nearby targets. As items are selected one at a time in sequence, the model accounts for how the underlying featural and spatial distributions of the remaining objects change.

Foraging strategy is frequently quantified by dependent measures such as average run length, which is the number of targets selected of a certain type before observers switch to selecting another target type. More broadly, switching between target types is intrinsically linked to relative proximity and to the number of targets of another category, both of which increase the likelihood of target switching. Clarke et al.'s (2022a, 2022b) model weights the relative contributions of parameters such as a tendency for target constancy, with biases for one target type over another. The generative model conceptualizes foraging as continuous sampling without replacement, since each consecutive target removal results in a change of the entire target distribution. Within a single trial, parameters are estimated for the probability of sticking to the same target type and a preference for a particular target category. The model also incorporates a proximity bias, estimating the likelihood that participants select targets close to the previously selected one. The extended multilevel model also incorporates individual differences in behavioural patterns.

However, these measures cannot be applied straightforwardly to continuous features without discrete target categories. The parameter of target type switching is not appropriate when the feature values of targets involve a continuous variable, such as colour. For the current study, we adapt and build upon this pre-existing model to allow the use of the continuous colour values in our foraging task.

## Current aims

We presented our observers with a multitarget display with numerous target exemplars within each trial, to closely represent a particular probability distribution of colours. The paradigm also allows for the sequential choice of target objects, a manipulation which enables the assessment of feature preferences over time. Representing a particular distribution of feature values onscreen is valuable due to evidence that subjective perceptual experience arises from sensory likelihood (Jazayeri & Movshon, 2007). Findings show that the neural units which are most informative are those which best discriminate between two conflicting stimuli, which suggests that templates intrinsically and necessarily take the statistics of a scene into account (Navalpakkam & Itti, 2007; Scolari & Serences, 2009). The fact that maximal information is provided by the largest change in signal contrast as opposed to the most active neuron tuned to an exact value, suggests that population coding is necessarily probabilistic (Averbeck et al., 2006; Tanrikulu et al., 2021).

Our approach involved applying a Bayesian multilevel model along the lines proposed in Clarke et al. (2022a, 2022b) which characterizes foraging as a generative sampling process without replacement, to foraging performance involving continuous colour values. We expected that the use of continuous target features would provide important insights into how selection of varied target types occurs, and to what degree observers are able to pick up information about the properties of target distributions. In another manipulation, the distribution properties were changed halfway through the experiment to determine how observers would respond to changing dynamics and how quickly they would learn a new colour distribution.

## Materials and methods

### Procedure

All participants took part in an experimental session lasting approximately 45 min that consisted of 12 blocks and a total of 48 foraging trials, with a break after each block. Stimulus sets were presented until a predetermined number of targets were selected, by clicking on them with a cursor controlled by an optical mouse. The active area on which a click was registered was fit exactly to the circumference of the target and distractor objects. Each trial ended after selection of 18 to 28 targets (randomized). Variable trial lengths were used to reduce predictability and maintain engagement—given that the task was designed to be relatively easy. The experimental session was preceded by one training block, involving foraging until all targets were selected. Participants were encouraged to respond as quickly and accurately as possible. They were provided with no additional instructions (so they did not know the identity of the targets when the experiment started), except that if a distractor was clicked, a tone indicated that this was an incorrect selection. This tone sounded for 1 s and participants could not continue with the task until it ended. After the 12th block, halfway through the experiment, participants were informed that the 'correct' colours had changed, but otherwise the task was identical. This was to prepare participants for the shift in distribution in the second half. The second distribution was introduced to track the emergence and adaptation of probabilistic templates over time***.*** There was a second training block prior to this new distribution, and afterwards the experiment continued as before.

### Stimuli

The colours of targets and distractors came from a linearised colour space consisting of 48 isoluminant hues to both (luminance: 30 cd/m^2^, all hues defined in Commission Internationale de l’Éclairage, CIE, 1931) where adjacent hues are separated by approximately one average just-noticeable-difference (JND; sourced from Witzel & Gegenfurtner, 2013, 2015). Witzel and Gegenfurtner (2013, 2015) established that JNDs for different hues are relatively consistent across observers; colours can therefore be separated into roughly equally discriminable steps. The use of perceptually linear colour space has been validated in ensemble perception studies (Maule & Franklin, 2015, 2016) and in prior studies on feature distribution learning (Chetverikov et al., 2017b; Hansmann-Roth et al., 2019).

**Fig. 1:**
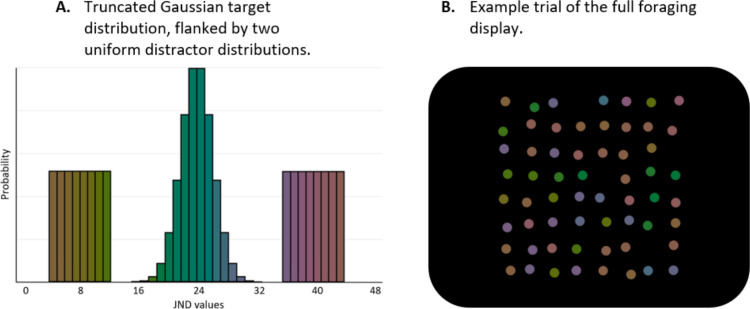
Example colour distributions that targets and distractors were drawn from, where **(A.)** is an example trial with targets centred on the JND value of 24. Two uniform distributions, from which distractor values are drawn, flank the truncated Gaussian target distribution. The two uniform distributions are both 3 JND's from the tails of the truncated Gaussian. A single exemplar trial **(B.)** shows the full display, including both targets and distractors. In this case, target values are also centred on the JND value 24, and disappear once selected. If distractors are selected, an auditory tone is played and the object disappears. (Colour figure online)

The targets were drawn from a truncated Gaussian distribution with a standard deviation of 4 JNDs and spanning a total of 16 JNDs, while the distractors were drawn from two uniform distributions, with identical range (16 JND range overall, eight JND for each distribution). The target values were drawn from a truncated Gaussian distribution for each separate trial, to be used as index values for the colour list. Figure 1A depicts both distributions.

The experiment was divided into two halves, with a different colour distribution for the first and the second 12 blocks. For each of these two halves, the colour distributions that the targets and distractors were drawn from were constant. For the second half, both distractor distributions and the target were shifted to the opposite end of the circular colour space, ±24 JNDs. The distance between the distractors and targets was constant at 3 JNDs, and variance was also constant, the only difference was the median of the distributions.

The foraging displays consisted of 30 distractors and 30 targets; all were disks that had a diameter of 0.5° of visual angle. All objects were jittered by 1° on an 8 × 8 invisible grid that subtended 11° × 11° (see Fig. 1B).

### Apparatus

Stimuli were displayed on a 24-in. LCD monitor Desktop PC on Windows 10, which was not colour calibrated, with a resolution of 1,920 × 1,080 and a refresh rate of 144 Hz, using PsychoPy (Version 2023.2.3; Peirce et al., 2019).

### Observers

Fifteen observers (eight men, *M*_age_ = 28.1 years) took part in the experiment. All observers were naïve to the goal of the study and had normal or corrected-to-normal vision. Sample size was determined in reference to previous foraging experiments (Á. Kristjánsson et al., 2020a, 2020b; T. Kristjánsson et al., 2020a, 2020b; Le et al., 2022) which had similar, and relatively small, numbers of participants due to the high volume of data provided within each individual foraging trial. Additionally, the modelling results revealed that the experiment was powered sufficiently well through the narrowness of the credible intervals and the sensitivity analyses to be performed. Prior to participation, all individuals' colour vision was screened using Ishihara colour plates to ensure normal colour perception. Every session was conducted in the same experimental room, with no overhead lighting, and with the same monitor and setup, to make the conditions as consistent as possible across participants. All gave written, informed consent prior to the experiment. The experiment was performed in accordance with the requirements of the local ethics committee and the Declaration of Helsinki.

### Model for continuous feature visual foraging

The model that we use to analyse the foraging data is a modification of the one proposed by Clarke et al. (2022b). The two spatial parameters, ρ_*δ*_ and ρ_*ψ*_, for distance between item selections and direction of travel, remain unchanged from the previous model as they account for intertarget distances and directions, that we expect to also be relevant in the present study. However, the two parameters that relate to preferences for selection of one target category over another have been adapted to consider only one target type, varying continuously by feature value. The current model re-defines the parameter denoting a preference for one target category over another, to a preference for targets of the mean colour value (the most probable value within the display). It also re-defines the preference for target selection of the same category as the previously selected target, to a preference for colours that are close in colour space. These adaptations accommodate the continuous feature values used in our display, allowing us to analyse the complex feature distribution.

The model treats foraging as a continuous process of sampling without replacement, where each item *i* has *p*_*i*_ probability of being selected as the next target, within a single trial. The probabilities of selection for the remaining targets are updated depending on the following four parameters (all continuous variables):

- ρ_*C*_: the 'colour' parameter, representing distance of a selected feature value from a prespecified feature value (in this case, the mean of the distribution). This is a feature tuning parameter, in which higher values indicate a greater preference for mean colour selection, and lower values indicate less preference for the JND distance between selected items and the mean colour. It can be thought of as the probability of selecting the mean, all else being equal.
- ρ_*S*_: the 'switch' parameter, representing a selection preference for previously selected feature values. This is a feature tuning parameter, in which higher values indicate a stronger preference for selecting values closer to the most recently selected item. For the first object selected within a trial, the probability *i* of selection of a specific colour value, is the proportion of that colour value present onscreen. For all subsequent selections, the probability depends on the identity of the previously selected target.
- ρ_*δ*_: the 'distance' parameter, which measures proximity, is more heavily weighted for the selection of items which are closer. It is the measure of the Euclidean distance between two objects, selected consecutively. The higher the value is, the greater the preference for items close to the previously selected item. This parameter is based on the parameter of the same name in Clarke et al. (2022b).
- ρ_*ψ*_: the 'direction' parameter, which measures relative angular distance in the direction of travel between two objects selected consecutively. This gives a value constrained between 0 and 1, where 0 indicates a target directly 'in front' of the previously selected target, 0.5 would indicate a 90° change of direction, and 1 would be to double back in space. This parameter was based on the parameter of the same name in Clarke et al. (2022b).

**Fig. 2:**
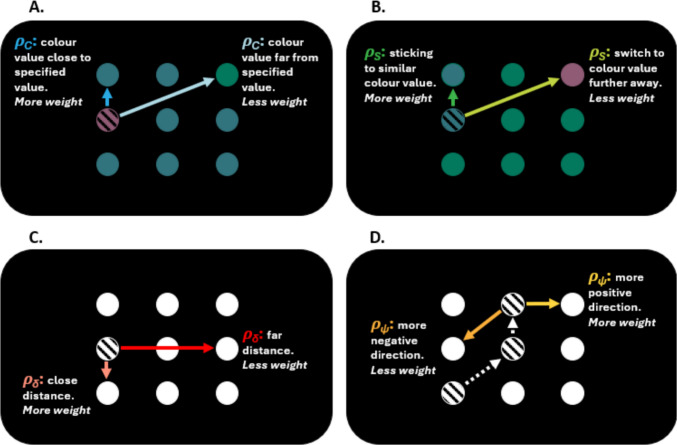
Illustrative foraging display, with hypothetical sequential selection weightings for the parameters of **(A.)** colour,** (B.)** switch,** (C.)** distance, and **(D.)** direction. (Colour figure online)

Figure 2 is a depiction of the four parameters within an illustrative foraging display, highlighting different hypothetical weightings associated with sequential selections, in spatial and feature distance. Figure 2A represents the colour parameter (ρ_*C*_); the shaded disc (containing horizontal lines) from which the arrows are pointing, depicts the specified colour value. The arrows represent two selection possibilities, and their associated weightings. The switch parameter (ρ_*S*_) is shown in Fig. 2B; the shaded disc depicts the previous colour selection within a trial, and the arrows point to two subsequent selection possibilities. Figure 2C relates to the distance parameter (ρ_*δ*_) and shows associated weightings for object selections of a smaller versus larger proximity, relative to the prior selection (the shaded disc). Lastly, the direction parameter (ρ_*ψ*_), seen in Fig. 2D, shows weightings for travel direction. The shaded discs and arrows represent previous selections (and therefore previous angular distance calculations).

The full formula for defining weights within the model is the following:

1 $$w(i)=e^{{-\rho}_cC(i)-\rho_cS(i,\;j-1)-\rho_\delta\delta(i,\;j-1)-\rho_\psi \psi(i,\;j-1,\;j-2)}$$

To calculate the likelihood of selection, let C(*i*) be the JND value of item *i* and S(*i*, *j* − 1) be the difference between previous item* j* and current value item *i*. For the spatial parameters, let δ(*i, j* − 1) be the proximity distance between previous and current value item *i* and ψ(*i, j* − 1, *j* − 2) be directional distance (calculating the direction from* j* − 2 to *j* − 1 and comparing this to the direction *j* − 1 to *i*).

Unit-level priors used for the model were:

2 $${\rho }_{c}\sim \mathrm{N}\left(\mathrm{0,01}\right)$$

3 $${\rho }_{s}\sim \mathrm{N}\left(\mathrm{0,0.1}\right)$$

4 $${\rho }_{\delta }\sim \mathrm{N}\left(\mathrm{15,5}\right)$$

5 $${\rho }_{\psi }\sim \mathrm{N}\left(\mathrm{0,1}\right)$$

Each fixed effect parameter is assumed to come from a normal distribution centered at the specified prior mean and standard deviation. Random effects across participants are modelled via a multivariate normal with a Cholesky factor and standard deviations. The random effects are transformed into correlated effects using a Cholesky factor and the fixed effects are added to random effects to get participant-specific parameters. The random effects have exponential priors for their standard deviations and an LKJ (probability distribution frequently used in Bayesian modelling, which ensures that correlation matrices remain positive) prior for their correlation matrix.

Weakly informative priors for ρ_*C*_ and ρ_*S*_ were chosen, since our main interest was whether parameters differed from 0. Each of these parameters follows a normal distribution with a mean of 0 and a variance of 0.1. The chosen values were based on model results from previous, related experiments (Clarke et al., 2022b). The two new parameters for colour and switches between targets were picked to account for a whole range of behaviours. For the random effects, we follow Lewandowski et al. (2009) and use an LKJ prior. We use an Exponential(5) distribution for the prior for all group-level variances.

This model is implemented in a multilevel framework, allowing each of the four parameters to vary between participants. It returns estimates of a full posterior probability distribution for each parameter. More information on the model can be found in the OSF depository.

R (R Core Team, 2024) and Stan (Stan Development Team, 2020) were used for model fitting. The fit was checked to ensure that r < 1.01 and trace plots were visually inspected to ensure convergence. No-U-Turn Sampler (NUTS) was used, at default settings.

## Results

### Behavioural results

The following section details our initial analysis of the descriptive statistics. These results will later be expanded upon with the newly adapted generative model, allowing us to directly break behavioural patterns down into independent biases (spatial and feature preferences) which influenced participant's target selection.

The average accuracy was 96 to 99% for correct selection of targets over distractors. Error rates were almost identical between the first and second distributions, indicating that participants were equally accurate in distinguishing between target and distractor objects in both halves of the experiment. Due to the high accuracy, distractor information was treated as a continuation of the feature value space (targets and distractors were not treated as discrete categories) and included in the modelling analyses.

**Fig. 3:**
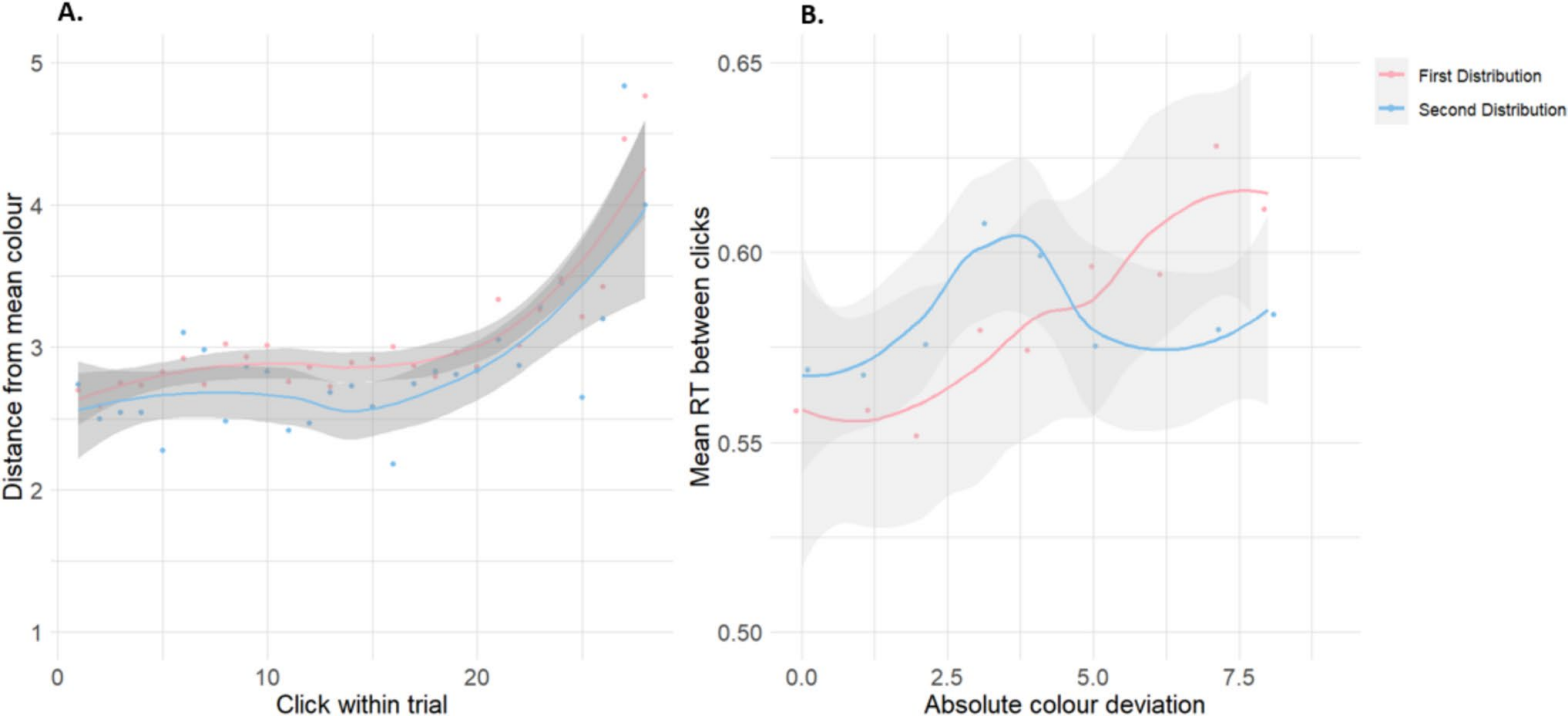
A. Mean absolute colour value selections for each click within a trial, aggregated across participants and separated between distributions. Locally weighted smoothing (LOESS method) was used to fit a curve to the data points, enabling a clearer interpretation of trends. **B.** Mean RT for absolute colour value selections aggregated across participants and trials, separated between distributions. The shaded regions represent the standard error around each line. (Colour figure online)

Initial analyses of selection order indicated that participants preferentially selected values closer to the mean, on average selecting values just under 3 JND values away. The order of selections within each trial provides us with information on which values were being prioritised; there was a slight trend towards selection of values further from the mean towards the latter half of the trial, seen in Fig. 3A. There was a very similar trend for both distributions; participants preferred selection of similar JND values up until the last third of the trial, where they were presumably forced to select colour values closer to the tails of the Gaussian target distribution, as they had depleted the colour values closer to the mean. Overall, participants appeared to have a strong preference towards selection of the more probable values onscreen, approximately 2 to 4 JND values from the mean.

Feature distribution learning is commonly operationalized via reaction time (RT) measurements, where more probable feature values lead to faster response time (Chetverikov et al., 2016; Hansmann-Roth et al., 2022, 2023; Pascucci et al., 2022). Therefore, we will briefly discuss the RT data here, since it is not included in the generative model. Figure 3B shows that responses were slower (calculated as time of current selection minus time of previous selection) for feature values farther from the mean, in the first distribution. However, this pattern was not evident in the second distribution, which showed more variability. This could possibly be interpreted as interference from the first distribution; participants were retaining a memory representation of the previous target colours which was impacting the learning of the new distribution (Sreenivasan et al., 2011).

**Fig. 4:**
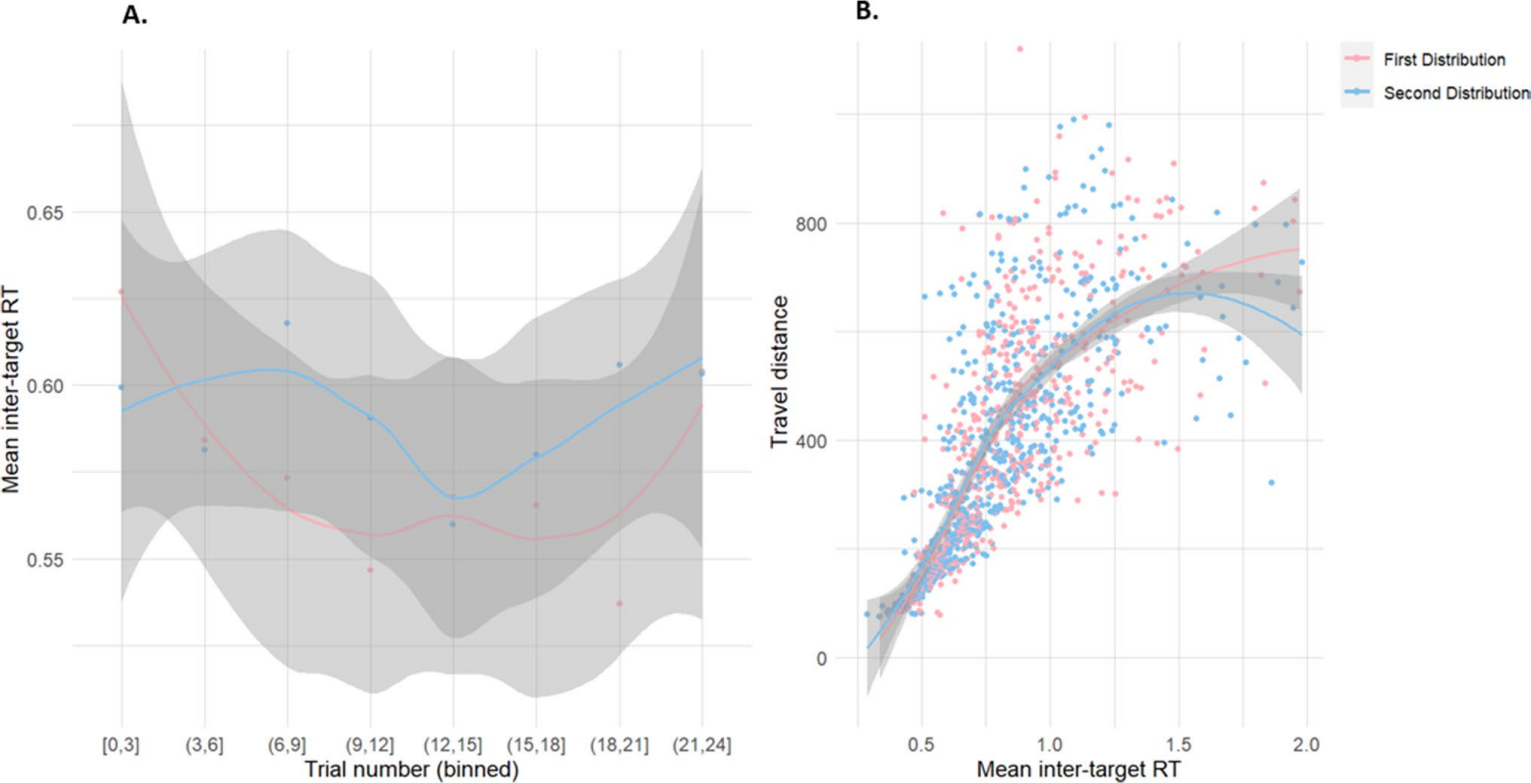
A. Mean intertarget selection times for targets aggregated across participants and split between the first and second distributions. Trials have been combined into bins of 3, for both distributions. The shaded region represents standard error. **B.** Relationship between travel distance and RT. Travel is measured as the Euclidean distance between target coordinates and mean inter-target selection time is time (in seconds) between subsequent target selections, aggregated across participants and split between the first and second distributions. The shaded regions represent the standard error. (Colour figure online)

As Fig. 4A shows, the mean inter-target RT within each condition, the time taken between subsequent selections of targets within a trial, remained relatively constant. The lack of any strong pattern might be due to a ceiling effect; the very small number of errors would indicate that participants were easily able to discern targets from distractors and would not have spent additional time searching for targets. The experiment was also relatively labour-intensive (requiring continuous search and selection for approximately 45 min) so fatigue may have played a role.

In Fig. 4B, we confirm the supposed relationship between travel distance (between subsequently selected target objects onscreen) and intertarget selection time. For both distributions, a strong trend exists between greater travel distance and slower RTs. This result reflects the time taken to bridge the physical distance between objects onscreen and may also indicate a preference of selection for more nearby objects (which will be discussed in the Modelling section). Travel distance was calculated as the Euclidean distance from the origin (0,0) to the point defined by the coordinates, rounded to the nearest integer.

### Modelling results

**Fig. 5:**
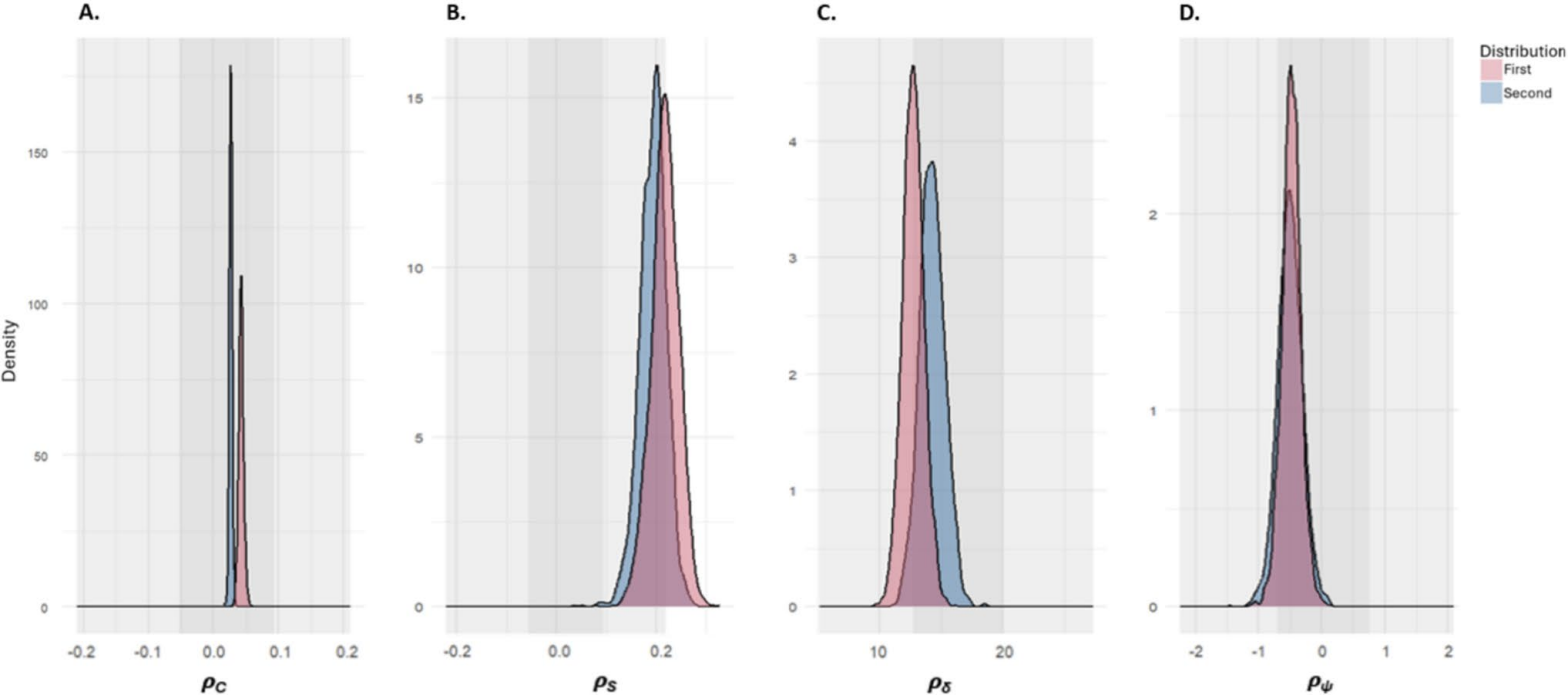
Posterior density estimates separated into ρ_C_; the colour parameter, ρ_S_; the switch parameter, ρ_δ_; proximity and ρ_ψ_; direction. First and second halves of the experiment are presented separately. The shaded region is the 97 Highest Posterior Density (HPD) interval of the prior distribution. (Colour figure online)

The most important result from the modelling analysis is that performance patterns reflected the distribution shape. Firstly, for the colour parameter in Fig. 5A, the posterior density estimates exhibit a positive shift away from zero. This indicates that participants were sensitive to the distance between the specified colour value (the mean value) and other colour values onscreen, preferencing selection of this colour. This accounts for any spatial biases (due to the way the model is constructed), and the inherent property of the display wherein the mean colour value was displayed at a higher frequency onscreen than the other values. This colour preference was more pronounced in the first distribution than in the second, which might suggest increased confidence in selecting feature values closer to the tails of the distribution, in the second half of the experiment.

Figure 5b, showing the switch parameter, demonstrates the relationship between the previous and current colour selection. The positive shift of the posterior density suggests that participants were more likely to select the same or similar colour value as the value that they had previously selected. The figure suggests that this effect was stronger than the effect for the colour parameter, although only by a small margin. The difference between the two distributions was negligible for this parameter.

The spatial model gives more weight to nearby targets (i.e., where distance is close to zero. see Fig. 5C). As can be seen by the high values, participants showed a very strong preference for selection of items within closer proximity. There was a small preference for reversing direction, as the negative shift from zero, in Fig. 5D indicates. This is in line with the general pattern of the spatial parameters in previous multitarget foraging analyses (Clarke et al., 2022a, 2022b).

**Fig. 6:**
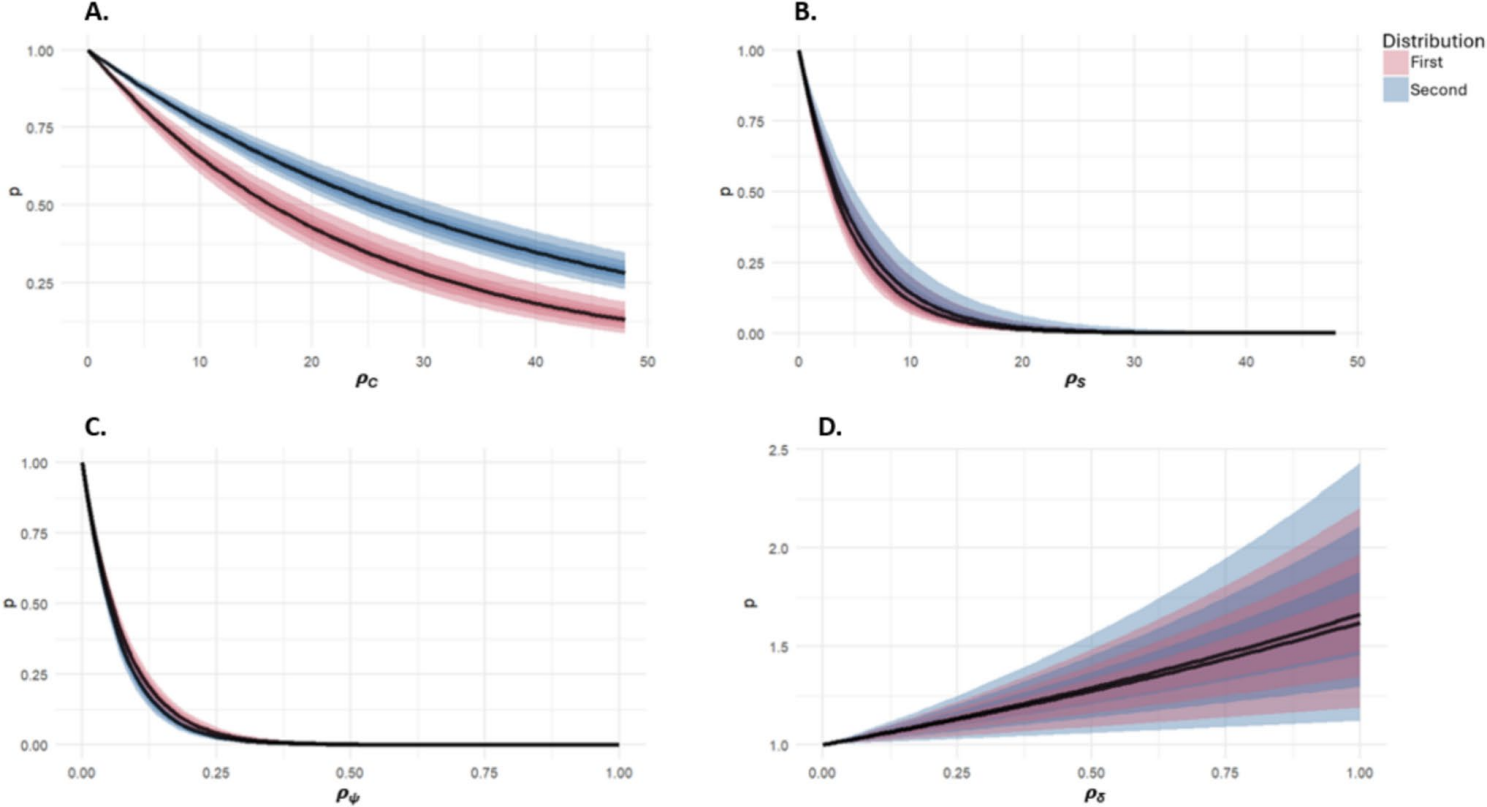
Posterior distributions for the continuous feature foraging. The shaded ribbons for the proximity and direction weighting indicate 53%, 89%, and 97% HPDIs, where the *y*-axis is probability weight and the *x*-axis is distance (colour, Euclidean, or angular). Parameters are presented separately. (Colour figure online)

The posterior density estimates were used to generate the probability curves in Fig. 6. Figure 6A shows the relationship between selection of the specified colour (the mean), and probability weight. From the slope of the curve, it can be seen that participants showed a preference for selecting more probable colour values; selecting values that were closer to the specified colour (over values that were less likely to be displayed onscreen). The fit of the slope maps onto the shape of the underlying Gaussian distribution that targets were drawn from, for both distributions. If there were simply a mean preference and the template encoded only the most probable value, but the application to filtering stimulus was noisy, the shape of the curve would show a much steeper fall-off. This preference appears to be stronger in the first than the second distribution, as can be seen by the steepness of the curve, as it falls away from zero (the mean value).

Figure 6B shows the relationship between the previous and current colour selection, and probability weight. The sharp curve suggests that participants were more likely to select the same or similar colour value as the value that they had previously selected, and that this was true for both distributions.

The spatial model gives more weight to nearby targets (i.e., where distance is close to zero), seen in Fig. 6C. Participants showed a very strong preference for selection of items within closer proximity. There was a small preference for reversing direction, as indicated by the positive skew of the highest posterior density interval (HPDI), seen in Fig. 6D. The HPDI is the shortest interval that encompasses a certain percentage of the posterior density.

**Fig. 7:**
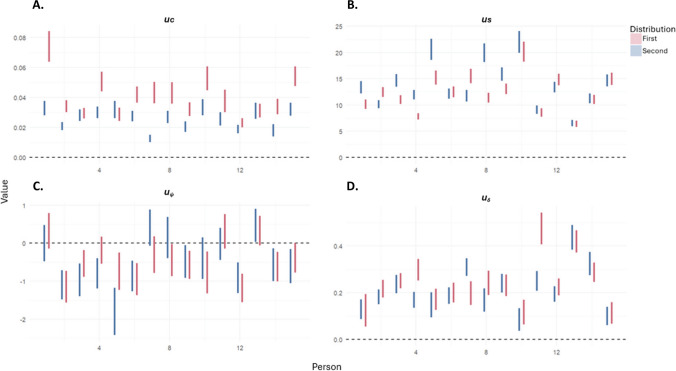
Individual differences for each of the parameters*,* where u_c_ is the colour parameter, u_s_ is the switch parameter, u_**δ**_ is proximity and u_**ψ**_ is the direction parameter. The shaded intervals are the 97% HPDI. The abscissa demonstrates individual participants. (Colour figure online)

As this model was fit using a multilevel framework, we can compare performance across participants. There was a fair amount of variability between participants, as Fig. 7 shows. Participants showed clear differences in their selection preferences; some preferring selection of objects based on proximity and others showing a greater preference for the feature value parameters. The two spatial parameters appear to be related: participants with a stronger preference to select nearby targets also have a more negative direction bias. This could be interpreted as a strategy to search within local patches compared with a more global scanning strategy.

Two other models were run, aside from the full model described above, for comparison. One of these models only included the proximity and direction parameters (ρ_δ_ and ρ_ψ_) and one only included the proximity, direction and switch (ρ_δ_, ρ_ψ_, and ρ_S_) parameters. After the models were fit to the data, a leave-one-out model comparison was performed, to calculate the posterior probabilities for each. This comparison is useful for assessing the predictive performance of a model; it is a direct comparison of models and can quantify how well each one predicts new data. In the output for the full model, the Monte Carlo *SE* (standard error) was small compared to the other uncertainties (0.1) and all Pareto *k* diagnostics were good (*k* < 0.7). We compared expected log predictive density (ELPD) for the three models. The ELPD is computed by making pairwise comparisons between each model and the model with the largest ELPD is always set at zero (difference between the preferred model and itself), which in this case was the full model. The difference in ELPD was much larger than the estimated standard error of the difference for the direction, proximity, and switch model (ELPD diff; −2,827.5 and *SE* diff; 26.1), and the direction and proximity model (ELPD diff; −4,375 and *SE* diff; 29.7), indicating that the full model has better predictive power than the other two. We concluded that the model that included parameters is superior and that the inclusion of feature value plays a large role in explaining foraging behaviour. The inclusion of the colour parameter is, in particular, vital to explaining participants' foraging behaviour; their sensitivity to the colour distance from the mean guided their selection preferences significantly.

## Discussion

Our main goal was to assess the learning of multitarget probabilistic distributions, and the mechanisms which guide selection during visual foraging. The visual system can learn probabilistic representations of the environment, by temporally integrating single exemplars of a target distribution (Hansmann-Roth et al., 2022). Here, we tested whether this finding can also be applied to multi-object distributions in a foraging task containing multiple target objects (drawn from the same feature distribution) on each trial. If true, this would rule out the possibility that only limited feature values of distributions are represented (e.g., the mean and variance of the distribution, as argued, for example, in summary statistics accounts; see Cohen et al., 2016; Corbett et al., 2023). For example, if target colour remained constant across trials, the feature learning might simply collapse to the learning of a distribution with a variance of zero, and by summary statistics accounts observers would learn the distribution mean.

Crucially, performance patterns reflected the distribution shape, suggesting a large degree of accuracy in probabilistic learning (see Chetverikov & Kristjánsson, 2022; Khvostov et al., 2025; Tanrikulu et al., 2021). Participants were more likely to select the mean colour value, in line with the encoding of summary statistics. We also found that participants were more likely to select more probable colour values, with diminishing likelihood of selection, the less probable a colour value was. If participants were solely aware of summary statistics, we should expect to only see a preference for the mean and for values adjacent to the mean, with colour values towards the tail of the distribution equally likely to be selected, despite their probability onscreen. The new generative model, developed for this study, accounted for the proximity and direction biases inherent in multitarget foraging tasks, such as the one used here. It also accounted for the inherent property of the display wherein the mean colour value was displayed most often onscreen and was more likely to be selected purely by chance.

A key interest for this study was to investigate the learning of feature distributions using a naturalistic paradigm. The study was designed to be representative of a naturalistic visual scene, in which feature distribution learning typically occurs (Chetverikov et al., 2016). Participants were not constrained in their selection choices, rewarded for selection of specific feature values, or given a time limit. For these reasons, it is interesting to observe that participants were more likely to select a colour value that was more probable in the visual scene. We were also interested in the emergence and adaptation of probabilistic templates over time—the reasoning behind the addition of a second distribution. There were, however, negligible differences in the selection patterns between the two. The only noticeable difference was a slightly stronger mean colour preference in the first distribution, and a proximity preference in the second which may reflect increased confidence in the task, in the latter half of the experiment. Participants may have been more comfortable selecting ‘riskier’ items (towards the tails of the distribution) and preferred to select closer items, to reduce manual cost. The similarity between the two distributions would suggest that learning of the distribution occurred at a similar rate, and that a similar selection strategy was employed. Coupled with the extremely low error rates, this would suggest a stability in the preferences exhibited.

A crucial benefit to our model is that it explores the dynamic process of decision-making for each selection, within each singular trial, opening new avenues of investigation on the nature of attentional templates and the learning of probability distributions. Participants showed competing preferences for sticking to the same colour values, and for selection of the exact colour value of the mean. This resulted in a 'drift' effect; participants preferred selection of the mean value above all other values, and then due to their preference for selecting nearby values (in the colour space) they drifted to the next most probable values, within the display. The basis of participants' representation of the underlying distribution could arguably be priming effects, which affected their decision-making. Classic repetition priming involves the cumulative changes in behavioural and neural response with repeated presentation of the same stimulus (Brinkhuis et al., 2020; Forster & Davis, 1984; Kristjánsson & Ásgeirsson, 2019; Maljkovic & Nakayama, 1994; Schacter & Buckner, 1998). In our study, participants were repeatedly primed to the more probable colour values, as these were the most frequent values presented onscreen. Coupled with a tendency towards selection of same values, an accurate representation of the underlying probability distribution emerged, judging from the behavioural responses of the participants.

These findings provide us with valuable information on selection strategies and the ways in which individuals navigate the process of decision-making, when presented with complex statistical evidence in information-rich environments. Participants favoured shorter travel distances, whilst still preferring to select more probable feature values. This suggests that participants were engaged in a trade-off between target certainty (confidence in their selection of a correct target, which increased as target probability became higher) and travel distance from one target to the next (Tagu & Kristjánsson, 2022b). Accuracy was overall very high, which may have contributed to the large proximity bias, as participants' certainty in their correct selection of targets would not have led to the motivation to seek out any particular feature values, such as to more strongly prefer the mean values. Additionally, since the mean preference and switch parameters are intrinsically interdependent, in a design where selected targets disappear, it is possible that greater mean preference would lead to a lower switch preference, and vice versa.

### Modelling decision-making

Decision-making is a key feature of the current task which makes the model that we applied to the observed order selection crucial in disentangling the implicit cognitive biases associated with multitarget displays. A strength of the model is its simplicity, utilizing only four parameters to characterize the relatively complex sequence of target selections within a foraging task, involving continuous target feature values. More traditional methods of analysis cannot reveal underlying cognitive biases inherently present in such a task. Furthermore, neglecting direction and distance tuning in a multitarget display would ignore the dynamic process of foraging in space. Additionally, a propensity for selecting values closer to the mean (the colour parameter) would not have been accounted for in traditional analyses which treat target values as discrete and categorical. The model's efficacy is most apparent for the switch parameter, particularly as a metric for the dynamic nature of multitarget foraging, within a single trial. Unlike the colour parameter, which assesses the absolute value difference of selected items from the mean of the distribution, the switch parameter considers whether participants are more likely to select values similar to those previously selected. By treating each selection instance as a separate observation, our approach reaches stable estimates of multiple biases very efficiently.

### Future developments

There are a number of potentially important factors that we have not considered in the current model. For one, it fails to allow for the modelling of inter-target selection times. The addition of a parameter for response times to this model would be beneficial, as an implicit measure of feature value preference, and is currently under development. A future model incorporating response time data could clarify the underlying biases and help to explain why the influence of feature value on selection behaviour was diminished in the second half of the experiment. It could be hypothesized, from the Bayesian analysis of order selection data, that this was due to a decreased proximity and direction bias in the latter half of the experiment, but further analysis would need to be done, to substantiate this. Another area of future development could be to adapt the direction tuning parameter to stimuli presented on a grid; in the current model the direction parameter was on a cardinal scale. The current experiment might also benefit from the analyses of other distribution types, such as targets from uniform or bimodal distributions, for comparison with the current Gaussian distribution. It would be interesting to compare the learning of these different distributions; whether they are learned with the same degree of fidelity as here, to test the limits of information pick-up in a foraging paradigm like the current one.

We note that a possible factor contributing to the mean preference, divorced from probabilistic learning, is that participants favoured the selection of feature values farthest from both distractor distributions. There is evidence that search templates contain ‘relational properties’ (Becker et al., 2025; Grössle et al., 2023) and that targets shift ‘off-veridical’ in response to the distractor context, when distractors are linearly separable from targets. In this way, representations exploit features in the distractor context that most predictably distinguish targets from distractors (Yu et al., 2024). Taking this into account, participants may have been unduly skewed towards the centre of the distributions, optimizing their template by tuning it away from both distractor distributions. This may have contributed to the pattern of the results, but notably, the findings from the switch parameter argue against this. Selection of the same or similar values was more probable, even when selections were toward the tail ends of the distribution. Additionally, the preference for selecting the same or similar values was stronger than the preference for selecting the mean colour. This is suggestive of an ongoing process of visual priming (Kristjánsson & Ásgeirsson), where participants adapted over time to the frequency of item values onscreen.

Another alternative explanation, connected to the previous point, is a preference for the median or the mode instead of the mean. We note, however, that the medians and modes were not always equal to the means, considering that the target values were drawn from a Gaussian distribution for each trial, and thus were not identical (in some trials, it is theoretically possible that there were no values from the median of the distribution). Tuning to a single median value is therefore inefficient, considering that a given trial may contain no exemplars from this part of the distribution.

Lastly, despite the use of a perceptual linear colour space, it is still possible that participants automatically ‘chunked’ colours into discrete colour categories and selected within these categories. Participants may have been able to group colour values closer to the mean into a single categorical representation or ‘segment’. Studies on ensemble perception have shown that although objects around us typically vary along continuous dimensions, we tend to perceive the objects using more discrete, categorical descriptions (Utochkin et al., 2018). It is possible that participants' representation of the distributions were coarser, but considering that the precise colour values onscreen differed on each trial, it would seem difficult to construct precise and stable colour categories.

## Conclusions

Probabilistic tuning of attentional templates seems the most tractable and logical way of facilitating visual foraging. Our results indicate that participants are capable of encoding complex and dynamic visual statistical information. The likelihood of selecting more probable colour values in the visual scene was higher, and this mapped onto the underlying colour probability distribution. The modelling results also account for other behavioural biases, such as proximity and direction preferences. Overall, the results suggest that target identification and selection is based on a trade-off between practical realities; travel distance, number of targets, and target preference (in this case, the more probable targets are preferred). Participants seek a 'selection balance' between proximity and priming, and show individual differences in their search strategy (Tagu & Kristjánsson, 2022a, b). The results indicate that probabilistic visual information is encoded with considerable fidelity, and is used to inform decision-making when observers forage for visual stimuli.

## Funding acquisition:

Á.K. and Á.G.Á.

**Investigation:** J.C.M.F.

**Methodology:** J.C.M.F., Á.K., and Á.G.Á.

**Project administration:** J.C.M.F., Á.K. and Á.G.Á.

**Resources:** J.C.M.F., Á.K. and Á.G.Á.

**Software:** J.C.M.F., A.C., and Á.G.Á.

**Supervision:** Á.K. and Á.G.Á.

**Validation:** J.C.M.F., Á.K., A.C., and Á.G.Á.

**Visualization:** J.C.M.F., Á.K., A.C., and Á.G.Á.

**Writing–original draft:** J.C.M.F.

**Writing–review & editing:** J.C.M.F., Á.K., A.C., and Á.G.Á.

## Data Availability

The processed trial-level data and analysis code for the generative model can be obtained from Open Science Framework (osf.io/kpqbf).

## Code availability

The custom code used for the analyses is available on Open Science Framework, and can be retrieved at osf.io/kpqbf
